# Awareness of Proton Pump Inhibitor Adverse Events and Treatment Pattern Change According to Physician Practice: A National Questionnaire Study in Korea

**DOI:** 10.3390/jpm14050529

**Published:** 2024-05-15

**Authors:** Yong Hoon Choi, Seung In Seo, Da Hyun Jung, Joon Sung Kim, Seung Young Kim, Hyun Chul Lim, Young Hoon Youn

**Affiliations:** 1 Department of Internal Medicine, Seoul National University Bundang Hospital, Seongnam 13620, Republic of Korea; 89796@snubh.org; 2 Division of Gastroenterology, Department of Internal Medicine, Kangdong Sacred Heart Hospital, Hallym University College of Medicine, Seoul 05355, Republic of Korea; 3 Division of Gastroenterology, Department of Internal Medicine, Severance Hospital, Yonsei University College of Medicine, Seoul 03722, Republic of Korea; jungdh@yuhs.ac; 4 Division of Gastroenterology and Hepatology, Department of Internal Medicine, Incheon St. Mary’s Hospital, College of Medicine, The Catholic University of Korea, Seoul 05355, Republic of Korea; 5 Department of Internal Medicine, College of Medicine, Korea University, Seoul 02842, Republic of Korea; ksymd@korea.ac.kr; 6 Department of Internal Medicine, Yongin Severance Hospital, Yonsei University College of Medicine, Yongin 16995, Republic of Korea; mdlhc97@yuhs.ac; 7 Department of Internal Medicine, Gangnam Severance Hospital, Yonsei University College of Medicine, Seoul 06273, Republic of Korea; dryoun@yuhs.ac

**Keywords:** proton pump inhibitor, adverse events, physician, practice, survey

## Abstract

Although adverse events of proton pump inhibitors (PPIs) have been reported, there are few studies on physicians’ perceptions. We aimed to investigate physicians’ awareness of PPI-related adverse events and changes in treatment patterns according to their practice. We conducted an online survey of physicians using a 15-item questionnaire. The survey queried respondents’ demographic information, PPI prescription patterns, perceptions, and concerns on the reported PPI-related adverse events. Concerns regarding the adverse events of PPI were assessed by dividing them into possibilities and medical causality. Of the 450 respondents, 430 were specialists, and 232 were gastroenterologists. A total of 87.8% of the respondents were generally or well aware of the adverse effects of PPI, 29.1% considered side effects when prescribing PPI, and 14.6% explained them to patients. Specialists were more aware of the side effects of PPI than general practitioners (*p* = 0.005), and gastroenterologists were more aware of the side effects of PPI than non-gastroenterologists (*p* < 0.001). However, gastroenterologists explained less to patients (*p* = 0.001) and preferred to reduce the dose of PPI rather than discontinue it. The adverse events that were recognized as having the highest probability of occurrence and strongest association with PPI use were bone diseases, *Clostridium difficile* infection, gastrointestinal infection, pneumonia, and interactions with anti-thrombotic drugs. Physicians’ awareness of PPI-related adverse events and treatment patterns differed significantly according to their positions and practice. Although a number of adverse events of PPIs were reported, physicians seem to accept their significance differently according to their specialty and practice patterns.

## 1. Introduction

Proton pump inhibitors (PPIs) are among the most widely prescribed medications worldwide [1,2]. PPIs decrease gastric acid production by inhibiting H^+^/K^+^ adenosine triphosphatase in parietal cells [3], and have proven effective in treating various diseases including gastroesophageal reflux disease (GERD) [4], peptic ulcer disease (PUD) [5], and functional dyspepsia [6]. Although PPI has been considered a fairly safe drug in the past few decades, numerous studies have reported its side effects including kidney disease [7], osteoporosis/bone fractures [8,9], dementia [10], *Clostridium difficile* (*C. difficile*) infection [11], and pneumonia [12]. In addition, the possibility of interaction between clopidogrel and PPI through cytochrome isoenzyme and gastric cancer (GC) has been controversial [13,14].

Although most studies reporting on the adverse events of PPI thus far are based on observational studies and may lack definitive evidence [15], continuous research results and media reports of these side effects have changed the perception of physicians prescribing PPI, and several questionnaire studies have been reported [16,17]. However, there are few surveys on physicians’ awareness and treatment patterns according to physician practice type, including many gastroenterologists. Hence, we aimed to investigate physicians’ perceptions of PPI adverse events and changes in treatment patterns according to their practices and hospital type. 

## 2. Materials and Methods

### 2.1. Study Design

This prospective observational online survey collected data in March 2023. Questionnaire items were created based on the adverse events of PPI, which have been frequently reported in previous studies [7,8,9,10,11,12,13,14]. The questionnaire was administered online to physicians at primary, secondary, and tertiary hospitals in Korea via e-mail. This study was reviewed and approved by the Seoul National University Institutional Review Board (B-2212-801-307), which waived the requirement for informed consent.

### 2.2. Study Participants

Approximately 700 subjects were invited to respond to the survey via e-mail, and the subjects were the members of the Korean Society of Neurogastroenterology and Motility and the institutional physicians and alumni of the study’s authors. This study targeted physicians who treat patients in clinical practice and prescribe PPI, regardless of their majors; medical students and non-physicians were excluded. Physicians who completed the survey were included in the study, and those who responded incompletely were excluded from the analysis.

### 2.3. Survey Instruments

The survey was conducted in Korean for Korean speakers. The questionnaire comprised 15 items, including multiple choice and multiple response questions (Supplementary File S1), modeled based on similar previous studies [16,17]. The survey queried respondents’ demographic information, PPI prescribing patterns, and perceptions and concerns regarding the reported side effects of PPI. Concerns regarding the side effects were addressed by dividing them into possibilities and medical causality.

### 2.4. Statistical Analysis

Responses to PPI prescription behavior, perception, and consideration of PPI side effects, and side effects that may be highly likely and medically causative were compared between groups according to age, major, training level, and clinical experience.

Chi-square and Fisher’s exact tests were used to analyze categorical variables, and Student’s *t*-test and Wilcoxon rank test were used to analyze continuous variables, as appropriate. Data were analyzed using IBM SPSS Statistics 25.0 (Chicago, IL, USA).

## 3. Results

### 3.1. Demographics of Respondents

The demographic information of the 450 physicians is summarized in Table 1. Approximately half of the respondents were gastroenterologists (232/450, 51.6%), 100 were majoring in internal medicine other than gastroenterology (100/450, 23.3%), and doctors with various majors were included. The numbers of physicians in primary, secondary, and tertiary medical institutions were similar.

The most frequently prescribed PPI was esomeprazole-rabeprazole-pantoprazole-lansoprazole, which was similar to the recent Korean PPI market share information [2]. The main indications for PPI prescription were GERD (87.1%), followed by PUD (61.3%), bone and joint diseases including rheumatoid diseases (19.1%), and a combination with anti-thrombotic drugs (including both anti-platelet agents and anti-coagulants, 18.0%). Most of the respondents (355/450, 78.9%) prescribed PPI for <2 months, whereas the rest (95/450, 21.1%) prescribed PPI for >three months.

### 3.2. Perception of Side Effects of PPI

Of the total survey respondents, 87.8% answered generally or were well aware of the adverse effects of PPI, and only 0.7% responded that they did not know at all. A total of 24.2% of respondents generally consider and 4.9% always consider side effects when prescribing PPI, whereas 14.6% answered that they frequently or consistently explained them to patients. Moreover, the rate of patients with concerns regarding the side effects of PPI was low.

Specialists were more aware of the side effects of PPI than general practitioners (*p* = 0.005), considered more when prescribing PPI (*p* = 0.032), and explained more to patients (*p* = 0.007). Gastroenterologists were more aware of the side effects of PPI than non-gastroenterologists (*p* < 0.001), although they explained them less to patients (*p* = 0.001). Senior doctors with > 8 years of clinical experience after training were more aware of the adverse effects of PPI than juniors (*p* < 0.001) and considered the adverse effects more when prescribing PPI (*p* = 0.008). Doctors working in advanced medical institutions were more likely to encounter patients with concerns regarding the adverse effects of PPI (*p* < 0.001) (Table 2).

### 3.3. Possibility of Occurrence and Medical Causality of Major Side Effects

**Figure 1:**
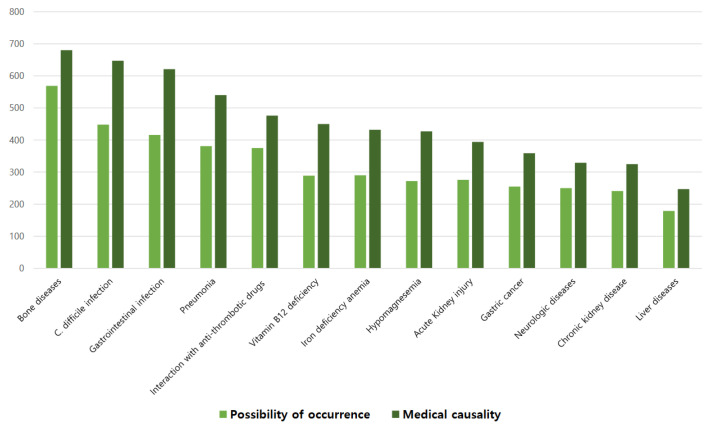
Responses to the likelihood and medical causality of known major side effects of PPI. *C. difficile*, *Clostridium difficile*.

The possibility of occurrence and medical causality of 13 side effects of PPI from previous reports [7,8,9,10,11,12,13,14] were rated from 0 points (no possibility or medical causality) to 4 points (high possibility or clear medical causality) for each item. The survey participants were asked to choose the adverse event that was most likely to occur with PPI use. They also chose the degree of causality of PPI use with the adverse event. Bone diseases, *C. difficile* infection, gastrointestinal infection, pneumonia, and interaction with anti-thrombotic drugs were reported as the most common adverse events associated with PPI use (Figure 1).

**Figure 2:**
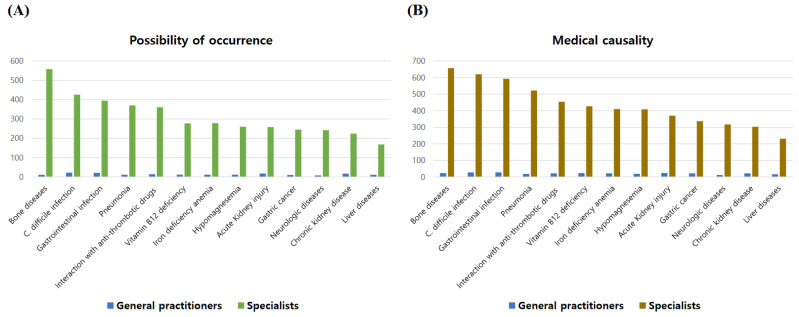

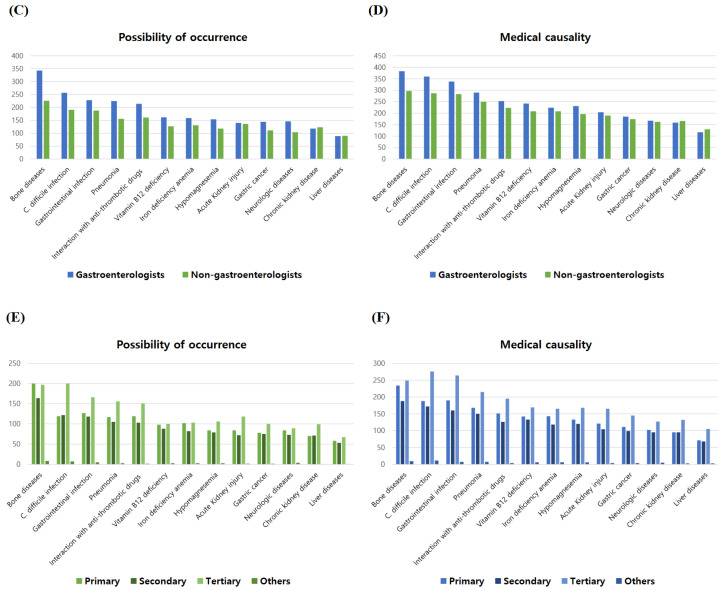
Responses to the likelihood and medical causality of known major side effects of PPI. (**A**,**B**) General practitioners versus specialists, (**C**,**D**) gastroenterologists versus non-gastroenterologists, (**E**,**F**) according to the size of the attending institution. *C. difficile*, *Clostridium difficile*.

Compared to general practitioners, specialists responded that the association of GC and liver diseases with PPI use was weaker, the possibility of occurrence of bone diseases was higher, and the possibility of occurrence and the association of acute renal injury and chronic renal disease with PPI use were lower and weaker. Gastroenterologists answered that the possibility and the association of *C. difficile* infection, pneumonia, and bone diseases with PPI use were higher and stronger compared with non-gastroenterologists. Doctors at tertiary medical institutions reported a higher possibility and stronger association of *C. difficile* infection and gastrointestinal infection with PPI use compared to the others. Nephrologists tended to report a higher possibility and stronger association of acute kidney injury with PPI use, although the number of respondents was small (Figure 2).

### 3.4. Prescription Changes Due to Consideration of Side Effects of PPI

Approximately 80% of all respondents answered that they would change the prescription considering the side effects of PPI, and most respondents answered that they would change it to on-demand therapy. Gastroenterologists considered changing prescriptions more often than non-gastroenterologists (consider change 86.2% vs. 73.9%, never change 13.8% vs. 26.1%, *p* = 0.008), and they preferred to reduce the dose of PPI, adjust the duration of administration, or change to on-demand therapy rather than discontinuing PPI administration or switching to H2 blockers when changing prescriptions (reducing the dose of PPI by 73.7% vs. 54.8%, *p* = 0.002) (Table 3).

## 4. Discussion

In this questionnaire study, we investigated the perception of PPI adverse events and changes in treatment patterns according to physicians’ practices such as the degree of training, specialty, clinical experience, and hospital type. Gastroenterologists, specialists, and experienced seniors were more aware of the side effects of PPIs than non-gastroenterologists, general practitioners, and juniors. Meanwhile, the adverse events of PPIs with a high probability of occurrence and medical causality were identified as bone diseases, followed by *C. difficile* infection, gastrointestinal infection, pneumonia, and interaction with anti-thrombotic drugs, which did not indicate statistical differences depending on the physician’s practice. In the treatment pattern, gastroenterologists explained the side effects of PPI to patients significantly more than non-gastroenterologists; however, they considered discontinuing PPI or changing to H2 blockers significantly less frequently than non-gastroenterologists.

In a previous survey study in the United States, more than half of PPI users had concerns regarding long-term side effects related to PPI use, and approximately one third of them changed their behavior based on concerns regarding PPI-related adverse events [16]. In addition, physicians demonstrated concerns on the side effects of PPI, that they responded aiming to de-escalate/de-prescribe PPIs [16], reducing the dose of PPI or changing to H2 blockers [18,19,20]. Another survey study has also revealed that most physicians are concerned of PPI-related adverse effects and changed their prescribing practices [17]. The main differences between our study and the previous two survey studies are the large proportion of GI specialists and detailed comparison depending on physician practice and hospital type. The increasing recognition of PPI adverse events by physicians and patients is attributed to the accumulation of publications on the possible complications of PPI, although conclusive evidence is lacking.

Hence, it is important to assess the medical causality between PPI and their various side effects. We asked separate questions on the possibility and medical causality of PPI-related side effects; however, many respondents answered positively to several side effects in terms of causality and the possibility of occurrence. The side effects of bone diseases and *C. difficile* infection were considered to be highly likely in our study, which was consistent with a previous survey study [17].

To date, the strength of the association between PPI and adverse events was weak, and residual confounding factors could not be ruled out in most studies [15,21]. Most studies reporting on the adverse events of PPI including those mentioned earlier are observational studies or case-control studies. For example, large-scale studies reporting the association between PPI and CKD [7], dementia [9], pneumonia [10], and GC were retrospective observational cohort studies, and the one reporting the association between PPI and hip fracture was nested case-control study [8]. Although these studies attempted to prove the association between PPI and its possible side effects through additional analyses using propensity score matching or time-varying model, it was impossible to completely exclude selection bias or other confounding factors. Vaezi et al. argued that factors such as strength, consistency, specificity, plausibility, and coherence should be considered when analyzing the side effects of PPI [15]. According to their meta-analysis, only studies on fundic gland polyps showed high strength, studies on bacterial enteric infection, hypomagnesemia, and fundic gland polyps showed consistency, and studies on hypomagnesemia, rhabdomyolysis, acute and chronic renal diseases, and dementia did not show plausibility. Reducing the administration of PPI in patients who do not need a prescription in consideration of the above side effects is crucial; however, the prescription of PPI should not be hesitant or discontinued in an excessively short period of time since not all studies to date on the side effects of PPI have proven medical causality. Previous studies have warned of excessive concern on the side effects of PPI or inappropriate discontinuation of PPI in necessary situations such as severe GERD or high risk for upper gastrointestinal bleeding [17,21].

In the current survey, physicians’ awareness of the side effects of PPIs did not lead to prescription changes or patient explanations. Expectedly, gastroenterologists, specialists, and experienced seniors were more aware of the side effects of PPIs than non-gastroenterologists, general practitioners, and juniors, respectively. This also suggests that physicians’ education is crucial regarding the benefits and risks of PPI prescriptions. However, even if physicians in tertiary institutions were more aware of the side effects, they explained them less to patients. In addition, gastroenterologists considered and explained the side effects of PPI to patients more often, although they considered discontinuing PPI or changing to an H2 blocker less often than non-gastroenterologists. This seems likely because most of the reported side effects based on research with low levels of evidence are not considered significant problems by gastroenterologists in clinical practice. Another possibility is that most of our study indications for PPI were GERD, and physicians might not have been able to discontinue PPI owing to patients’ symptom control. Therefore, the side effects of PPI need to be recognized. However, not all side effects of PPI have a causal relationship.

This study has several strengths. First, we included a considerably larger number of doctors than previously reported survey studies, with a significant number of experts in gastroenterology and a similar number of non-gastroenterologists. In the current survey, physicians’ awareness of the side effects of PPIs did not lead to prescription changes or patient explanations, which is different from the previous two survey studies. In addition, opinions on the possibility and medical causality between general practitioners and specialists were different, and gastroenterologists preferred to reduce the dose of PPI rather than discontinuation of PPI or change to H2 blocker. This is because the proportion of specialists who responded to this questionnaire was high, and in Korea the proportion of specialists among all doctors is high at over 70%, and they are thought to be relatively familiar with PPI. Additionally, the distribution of doctors in primary, secondary, and tertiary medical institutions is even. Finally, we compared physicians’ perceptions and treatment patterns according to physician practice and hospital type.

Nevertheless, this study also has some limitations. First, >95% of the respondents were specialists, and the proportion of general practitioners was very low. Second, most respondents were internal medicine specialists; however, the proportion of specialists other than gastroenterologists, such as nephrologists, endocrinologists, and cardiologists, was low. To compensate for these limitations, a survey of experts from various specialized fields is required. Third, we did not include patients’ perceptions of PPI adverse events.

## 5. Conclusions

In conclusion, physicians’ perceptions of PPI adverse events were high; however, the changing treatment patterns significantly differed according to physician practices. Bone diseases and *C. difficile* infection were the most significant adverse events recognized. Although a number of adverse events of PPIs were reported, physicians seem to accept their significance differently according to their specialty and practice patterns.

## Figures and Tables

**Table 1:** The demographic information of responded physicians.

**Variable**	*N* = 450 (%)					
**Age**	42.60 ± 7.53 (26–70)					
**Clinical experiences after obtaining specialist qualification**	9.47 ± 7.93 (0–37)					
**Sex**	Male		313 (69.6)			
	Female		136 (30.2)			
	Unanswered		1 (0.2)			
**Affiliated medical institutions**	Primary		152 (33.8)			
	Secondary		126 (28.0)			
	Tertiary		166 (36.9)			
	Military service/public institutions/others		6 (1.3)			
**Degree of training**	General practitioners	20 (4.4)				
	Specialists	430 (95.6)	Internal medicine	332 (73.8)	General	53 (11.8)
					Gastroenterology	232 (51.6)
					Pulmonology	5 (1.1)
					Cardiology	11 (2.4)
					Nephrology	11 (2.4)
					Infectology	4 (0.9)
					Endocrinology	9 (2.0)
					Hemato-oncology	5 (1.1)
					Rheumatology	1 (0.2)
					Allergy	1 (0.2)
			Pediatrics	5 (1.1)		
			Orthopedics	9 (2.0)		
			Otolaryngology	2 (0.4)		
			Anesthisiology	6 (1.3)		
			Neurology	2 (0.4)		
			Neurosurgery	1 (0.2)		
			General surgery	2 (0.4)		
			Cardiothoracic surgery	1 (0.2)		
			Familial medicine	11 (2.4)		
			Ophthalmology	1 (0.2)		
			Psychiatry	1 (0.2)		
			Emergency medicine	2 (0.4)		
			Obstetrics and gynecology	1 (0.2)		
			Radiology	1 (0.2)		
			Plastic surgery	2 (0.4)		
			Unanswered	71 (15.8)		
PPIs Preferred for Prescription *	Omeprazole		42 (9.3)			
	Esomeprazole		312 (69.3)			
	Pantoprazole		166 (36.9)			
	Lansoprazole		158 (35.1)			
	Rabeprazole		194 (43.1)			
	Dexlansoprazole		71 (15.8)			
	PPI+NSAIDs combination		29 (6.4)			
	Others		8 (1.8)			
**Number of prescriptions** **(number of patients per week)**	<5		81 (18.0)			
	6–25		146 (32.4)			
	26–50		120 (26.7)			
	51–100		73 (16.2)			
	>100		30 (6.7)			
Indications for PPI prescription *	GERD		392 (87.1)			
	PUD		276 (61.3)			
	Osteoarthritis, rheumatoid disease		86 (19.1)			
	With anti-thrombotic drugs **		81 (18.0)			
	Helicobacter pylori eradication		3 (0.7)			
	With steroids		5 (1.1)			
	Other diseases		3 (0.7)			
**Prescription period**	Within 1 month		203 (45.1)			
	1–2 months		152 (33.8)			
	3–4 months		67 (14.9)			
	5–6 months		10 (2.2)			
	Over 6 months		18 (4.0)			

PPI, proton pump inhibitor; GERD, gastroesophageal reflux disease; PUD, peptic ulcer disease. * Multiple responses allowed. ** Anti-thrombotic agents include both anti-platelet agents and anti-coagulants.

**Table 2:** Perception of side effects of proton pump inhibitors.

		Overall *N* = 450	Degree of Training			Majoring Gastroenterology			Clinical Experiences			Affiliated Medical Institutions				
			General Practitioners *n* = 20 (%)	Specialists *n* = 430 (%)	*p*-Value	Gastroenterologists *n* = 232 (%)	Non-Gastroenterologists *n* = 218 (%)	*p*-Value	<8 Years *n* = 194 (%)	≥8 Years *n* = 148 (%)	*p*-Value	Primary *n* = 152 (%)	Secondary *n* = 126 (%)	Tertiary *n* = 166 (%)	Others *n* = 6 (%)	*p*-Value
Awareness of side effects	Not knowing at all	3 (0.7)	0 (0.0)	3 (0.7)	0.005	0 (0.0)	3 (1.4)	<0.001	1 (0.5)	2 (1.4)	<0.001	1 (0.7)	1 (0.8)	1 (0.6)	0 (0.0)	0.057
	Not familiar with	52 (11.5)	7 (35.0)	45 (10.5)		9 (3.9)	43 (19.7)		27 (13.9)	14 (9.5)		10 (6.6)	16 (12.7)	26 (15.7)	0 (0.0)	
	In general knowledge	301 (66.9)	12 (60.0)	289 (67.2)		151 (65.1)	150 (68.8)		146 (75.3)	86 (58.1)		116 (76.3)	86 (68.3)	94 (56.6)	5 (83.3)	
	Well-informed	94 (20.9)	1 (5.0)	93 (21.6)		72 (31.0)	22 (10.1)		20 (10.3)	46 (31.1)		25 (16.4)	23 (18.3)	45 (27.1)	1 (16.7)	
Consideration of side effects when prescribing	Never	54 (12.0)	6 (30.0)	48 (11.2)	0.032	24 (10.3)	30 (13.8)	0.084	32 (16.5)	12 (8.1)	0.008	18 (11.8)	15 (11.9)	19 (11.4)	2 (33.3)	0.844
	Sometimes	265 (58.9)	10 (50.0)	255 (59.3)		129 (55.6)	136 (62.4)		116 (59.8)	92 (62.2)		90 (59.2)	74 (58.7)	99 (59.6)	2 (33.3)	
	Usually	109 (24.2)	2 (10.0)	107 (24.9)		64 (27.6)	45 (20.6)		43 (22.2)	33 (22.3)		37 (24.3)	31 (24.6)	40 (24.1)	1 (16.7)	
	Always	22 (4.9)	2 (10.0)	20 (4.6)		15 (6.5)	7 (3.2)		3 (1.5)	11 (7.4)		7 (4.6)	6 (4.8)	8 (4.8)	1 (16.7)	
Explain side effects to the patient	Never	142 (31.6)	13 (65.0)	129 (30.0)	0.007	53 (22.8)	89 (40.8)	0.001	69 (35.6)	46 (31.1)	0.463	40 (26.3)	40 (31.7)	59 (35.5)	3 (50.0)	0.051
	Sometimes	242 (53.8)	4 (20.0)	238 (55.3)		139 (59.9)	103 (47.2)		101 (52.1)	80 (54.1)		90 (59.2)	64 (50.8)	87 (52.4)	1 (16.7)	
	Usually	51 (11.3)	2 (10.0)	49 (11.4)		31 (13.4)	20 (9.2)		21 (10.8)	16 (10.8)		16 (10.5)	21 (16.7)	13 (7.8)	1 (16.7)	
	Always	15 (3.3)	1 (5.0)	14 (3.3)		9 (3.9)	6 (2.8)		3 (1.5)	6 (4.1)		6 (3.9)	1 (0.8)	7 (4.2)	1 (16.7)	
Experiencing patients who are concerned about side effects	Never	185 (41.1)	17 (85.0)	168 (39.1)	0.001	60 (25.9)	125 (57.3)	<0.001	91 (46.9)	54 (36.5)	0.199	49 (32.2)	55 (43.7)	78 (47.0)	3 (50.0)	<0.001
	Rarely	228 (50.7)	3 (15.0)	225 (52.3)		147 (63.4)	81 (37.2)		85 (43.8)	82 (55.4)		90 (59.2)	64 (50.8)	72 (43.4)	2 (33.3)	
	From time to time	34 (7.5)	0 (0.0)	34 (7.9)		24 (10.3)	10 (4.6)		16 (8.2)	11 (7.4)		12 (7.9)	7 (5.6)	15 (9.0)	0 (0.0)	
	Often	3 (6.7)	0 (0.0)	3 (0.7)		1 (0.4)	2 (0.9)		2 (1.0)	1 (0.7)		1 (0.7)	0 (0.0)	1 (0.6)	1 (16.7)	

**Table 3:** Change prescriptions considering side effects of proton pump inhibitors.

	Overall *N* = 450 (%)	Gastroenterologists *n* = 232 (%)	Non-Gastroenterologists *n* = 218 (%)	*p*-Value
Never	89 (19.8)	32 (13.8)	57 (26.1)	0.008
Rarely	277 (61.5)	156 (67.2)	121 (55.5)	
From time to time	62 (13.8)	31 (13.4)	31 (14.2)	
Often	22 (4.9)	13 (5.6)	9 (4.1)	
	**Overall** *N* = 633 * **(%)**	**Gastroenterologists** ***n* = 354** **(%)**	**Non-Gastroenterologists** ***n* = 279** **(%)**	***p*-Value**
Dose reduction of PPI	110 (17.4)	68 (19.2)	42 (15.1)	0.002
Adjust the duration of the PPI	68 (10.7)	46 (13.0)	22 (7.9)	
Change to on-demand therapy	236 (37.3)	147 (41.5)	89 (31.9)	
Discontinuation of PPI	99 (15.6)	40 (11.3)	59 (21.1)	
Switch to H2 blocker	120 (19.0)	53 (15.0)	67 (24.0)	

PPI, proton pump inhibitor. * Multiple responses allowed.

## Data Availability

Data is available upon request.

## Supplementary Materials

The following supporting information can be downloaded at: https://www.mdpi.com/article/10.3390/jpm14050529/s1, File S1: The questionnaire in the study.
