# Supplementary material for: Awareness of Proton Pump Inhibitor Adverse Events and Treatment Pattern Change According to Physician Practice: A National Questionnaire Study in Korea
Source: J Pers Med. 2024 May 15;14(5):529. doi: 10.3390/jpm14050529 (PMC11122597; doi:10.3390/jpm14050529)
Supplement: Supplementary file 1 [file jpm-14-00529-s001.zip › jpm-2963142-supplementary.pdf]

1. Please record your age.

2. Please select your gender.

① Man/male ② Woman/female ③ Other / Do not want to respond

3. Please select the type of institution you are currently working for.

① Primary clinic ② Secondary hospital ③ Tertiary hospital ④ Others (military service / public institutions)

4. Please select your training level. If you are a professional, please record your specialized subject and year of acquisition.
4-1. If you are an internal medicine specialist, please record the detailed subspecialty.

5. Please select the Proton Pump Inhibitor (PPI) you usually prescribe. (Multiple choices available)

① Omeprazole ② Esomeprazole ③ Pantoprazole ④ Lansoprazole ⑤ Rabeprazole ⑥ Dexlansoprazole ⑦ PPI + NSAIDs compounds

6. How many patients do you prescribe proton pump inhibitor (PPI) on average per week?

① < 5 ② 5-25③ 26-50 ④ 51-100 ⑤ > 100

7. In what diseases do you usually prescribe proton pump inhibitor (PPI)? (Multiple choices available)

① Gastroesophageal reflux disease

② Peptic ulcer disease

③ Bone and joint diseases

④ Prevention of gastrointestinal bleeding in the treatment of antiplatelet drugs and anticoagulants

⑤ Others

8. How long is the Proton Pump Inhibitor (PPI) prescribed in practice (maximum duration)?

① < 1 month ② 1-2 months ③ 2-4 months ④ 4-6 months ⑤ > 6 months

9. Are you aware of the adverse effects of proton pump inhibitor (PPI)? (   )

① Not knowing at all ②Not familiar with ③ In general knowledge ④ Well-informed

10. How much do you consider possible adverse effects when prescribing proton pump inhibitor (PPI)?

① Never ② Sometimes ③ Usually ④ Always

11. Do you explain to the patient the risk of side effects when prescribing Proton Pump Inhibitor (PPI)?

① Never ② Sometimes ③ Usually ④ Always

12. Has the patient ever expressed concern about adverse effects when prescribing proton pump inhibitor (PPI)?

① Never ② Rarely ③ From time to time ④ Often

13. Please indicate how much you are concerned about each of the following items that have been reported as side effects of Proton Pump Inhibitor (PPI).

	**0 Never**	**1 Slightly**	**2 Somewhat**	**3 Considerably**	**4 Highly**
Gastric cancer					
Liver diseases					
Clostridium difficile infection					
Gastrointestinal infection					
Pneumonia					
Bone diseases (osteoporosis,					

fracture, Vitamin D deficiency)					
Neurologic diseases (Cognitive impairment, dementia)					
Vascular diseases (interaction with antithrombotic agents)					
Acute kidney injury (interstitial nephritis)					
Chronic kidney disease					
Iron deficiency anemia					
Vitamin B_{12} deficiency					
Hypomagnesemia					

14. Please indicate how much you think there is a medical causal relationship with the administration of PPI in each of the following items that have been reported as side effects of Proton Pump Inhibitor (PPI).

	**0 Never**	**1 Slightly**	**2 Somewhat**	**3 Considerably**	**4 Highly**
Gastric cancer					
Liver diseases					
Clostridium difficile infection					
Gastrointestinal infection					
Pneumonia					
Bone diseases (osteoporosis, fracture, Vitamin D deficiency)					
Neurologic diseases (Cognitive impairment, dementia)					
Vascular diseases (interaction with antithrombotic agents)					
Acute kidney injury (interstitial nephritis)					
Chronic kidney disease					
Iron deficiency anemia					
Vitamin B_{12} deficiency					
Hypomagnesemia					

15. Do you consider discontinuing or changing the prescription due to side effects of Proton Pump Inhibitor (PPI)?

① Never ② Sometimes ③ Usually ④ Always

15-1. How do you usually change your prescription if you are concerned about the side effects of long-term administration?

① PPI dose reduction (e.g., Esomeprazole 40 mg Q.D. to 20 mg Q.D.)

② Adjust the interval between PPIs (e.g., every 2 or 3 days)

③ Change to on-demand therapy

④ Switch to H2 receptor blocker

⑤ Discontinuation of medication
